# Bridging the gap between community health workers’ digital health acceptance and actual usage in Uganda: Exploring key external factors based on technology acceptance model

**DOI:** 10.1371/journal.pdig.0001099

**Published:** 2025-11-19

**Authors:** Miiro Chraish, Chisato Oyama, Yuma Aoki, Ddembe Andrew, Monami Nishio, Shoi Shi, Hiromu Yakura

**Affiliations:** 1 MobiKlinic Foundation, Kampala, Uganda; 2 Institute of Biochemistry II, Goethe-Universität Frankfurt am Main, Frankfurt am Main, Germany; 3 Fvital Inc., Tokyo, Japan; 4 National Center for Child Health and Development, Tokyo, Japan; 5 International Institute for Integrative Sleep Medicine, University of Tsukuba, Tsukuba, Japan; 6 Center for Humans and Machines, Max-Planck Institute for Human Development, Berlin, Germany; Universitat Oberta de Catalunya, SPAIN

## Abstract

Community health systems are poised to play a prominent role in achieving universal health coverage in low- and middle-income countries, as demonstrated during the COVID-19 pandemic response. The advent of health information technology has provided an opportunity to optimize the community health space and improve efficiency. However, there is limited knowledge about the acceptance and usage of health information technology among community health workers, a prerequisite for scaled implementation. This study aimed to use the technology acceptance model (TAM) to predict the acceptance and usage of health information technology among CHWs, identify external factors, and understand the impact on community health systems. Specifically, we conducted semi-structured interviews with 170 community health workers who were recruited through both convenience and snowball sampling. We then performed response coding and cross-tabulation, correlation, and regression analysis. As a result, the TAM effectively predicted CHWs’ behavioral intention to use digital health tools. However, actual usage was not well predicted, and there was a mismatch between high behavioral intention and low actual usage. Access to smartphones emerged as a major determinant of actual usage, overshadowing other variables in the TAM. In conclusion, while CHWs show strong acceptance of digital health tools, structural barriers, particularly limited access to smartphones, hinder their actual use. These findings highlight the importance of addressing infrastructural inequities to enable the effective and equitable digitization of community health systems.

## Author summary

Community Health Workers (CHWs) are the foundation of healthcare delivery in many low- and middle-income countries, including Uganda. As digital health technologies expand globally, governments and organizations are working to digitize community health systems to improve efficiency and access to care. However, despite widespread enthusiasm, many digital health initiatives fail to move beyond pilot phases, often because actual technology use among CHWs remains low even when acceptance is high. In this study, we applied the Technology Acceptance Model (TAM) to understand why CHWs in Uganda accept digital health tools but do not always use them in practice. We conducted semi-structured interviews with 170 CHWs across urban and rural communities. While most CHWs expressed strong willingness and positive attitudes toward using digital tools, our analysis revealed that limited access to smartphones was the main barrier preventing regular use, especially when sharing smartphones with acquaintances is common. Our findings show that improving CHWs’ access to smartphones and addressing structural inequalities are essential for successful and equitable digital transformation of community health systems. Efforts to digitize healthcare must therefore go beyond training and motivation to include infrastructure equity and sustained support mechanisms that empower CHWs to use digital health tools effectively.

## Background

In Uganda and other low-income countries, Community Health Workers (CHWs) play a central role in delivering basic health education, promoting disease prevention, and facilitating referrals at the village level [1,2]. They typically receive short-term training (5–10 days) and are not formally certified or licensed like doctors or nurses. Unlike medical professionals who diagnose and treat complex conditions, CHWs—locally referred to as Village Health Teams (VHTs)—focus on community outreach and primary care, serving as a critical bridge between households and the formal health system [3,4].CHWs have proven to be valuable constituents of health systems in low- and middle-income countries [5,1]. They have demonstrated the ability to offer much-needed primary health services to vulnerable populations such as children under 5 and pregnant women in remote and underserved communities [5,2,6]. Significantly, the paradigm has shifted from proving their relevance to unlocking their full potential as suitable buffers to healthcare inadequacies such as poor doctor-to-patient ratios and inequitable distribution of health services, especially in rural areas [3,7–12].

CHWs were vital in the fight against COVID-19 as contact tracers and community mobilizers for mass vaccination campaigns [4,13–16]. The success stories of community health systems during the COVID-19 pandemic reignited prior conversations about extending the scope of work, formal recognition, and apportioning budget votes for CHWs to cater to their remuneration [17–19]. It also called for acceleration of efforts towards optimizing community health systems [4,14,18].

The advent of mobile health technologies has yet to provide an opportunity for optimizing community health systems through improving health records management, supportive supervision, monitoring and evaluation and planning for community health systems [20–23]. This is poised to enhance the efficiency of individual CHWs and community health systems and ultimately ensure universal access to quality primary healthcare [24–26].

The government of Uganda through the Ministry of Health (MoH) has embarked on a concerted journey to foreclose on digitization of all CHWs in Uganda as a way of improving output, accountability and support supervision cost-effectively [27,28]. This has been advanced through an effort of partnerships with nongovernment and private entities to spur innovation in mobile health space while ensuring integration and interoperability [29–32]. Eventually, various digital community health platforms have been developed and piloted with an end goal of scaling regionally or countrywide. Despite the marvel of the various digital tools, few have gone beyond piloting, and those that did failed to achieve full potential and scalability [33,34]. This is hinged on the fact that there is limited knowledge about the acceptability, adoptability, cost effectiveness and usability of emerging technologies by CHWs in last-mile areas [35,36].

Consequently, Uganda launched her first 5-year National Community Health Strategy in February 2023 [37] followed by the Uganda Health Information and Digital Health Strategic Plan in May 2023 [38]. These offer guidance on the dual alignment of community health and digital health in the interest of public health. The sequence of these events underscores the notion that community health systems and digital health will play a large, intertwined role in achieving sustainable universal health coverage in the country [24,25,39,40]. It is upon this background that the MoH and partners have piloted the electronic community health information system (eCHIS) and plan to scale it countrywide [41] as the standard for digital community health tools.

However, prior interventions in public health services in Uganda, such as user fees’ introduction, village health team (VHT) program scaling and health service decentralization [42–45], have shown that insufficient knowledge before scaled implementation often results in underwhelming results, despite the massive system adjustments and significant cost bearing. On the other hand, the inevitability of emerging technologies in healthcare and associated enormous installation and maintenance costs leave no room for faltering right from the early implementation stages. This further emphasizes the need to build a good knowledge foundation to provide guidance on relevant variables, moderators and mediators in the different healthcare niches where technology is being implemented [40]. Most related studies in similar settings have assessed technology acceptance among more literate, better trained, compensated, and regulated formal healthcare professionals [46,47], while Campbell et al. [48] focused on the end-users’ acceptance of short message-based intervention in Uganda. Here, these professionals and end-users are fundamentally distinct from semi-literate, informal CHWs, who operate at the interface between informal community structures and the formal health system [42,43,46,47]. The confluence of all these realities and the scarcity of evidence to guide the uptake of digital tools among CHWs necessitated this study. The study aimed to predict technology acceptance among last mile CHWs using the Technology Acceptance Model (TAM), with a keen emphasis on discovering external (exogeneous variables) factors, establishing the impact of digital health tools on community health systems and understanding the social-technical aspects of digital health among community health workers.

## Theoretical framework and hypothesis development

### Technology acceptance model

We used TAM to hypothesize the acceptance and usage of digital tools among CHWs working either in Buikwe or Kampala. TAM is built off the theories of reasoned action (TRA) [49,50] and planned behavior (TPB) [51]. People’s acceptance and behavior towards information technology (IT) in healthcare and other sectors has often been explained and predicted using TAM, which was originally proposed by Davis in the 1980s [52].

TAM has received extensive application in studies exploring the acceptance and use of various health information technologies among patients and healthcare professionals [53–56], as well as personal computers and mobile applications [57]. TAM has proven to be a standard predictor of technology acceptance and usage among various populations. Nonetheless, TAM has not been applied to predict CHWs’ acceptance of digital health tools in Uganda and has seen limited use among similar populations in other related jurisdictions. Given the early stage of technology deployment in Uganda’s community health systems, and the literacy levels of CHWs, TAM provided a more appropriate and insightful framework for guiding survey design. Our study therefore extends TAM into the under-researched context of CHWs, who operate at the community–health system interface in low-resource settings.

**Fig 1:**
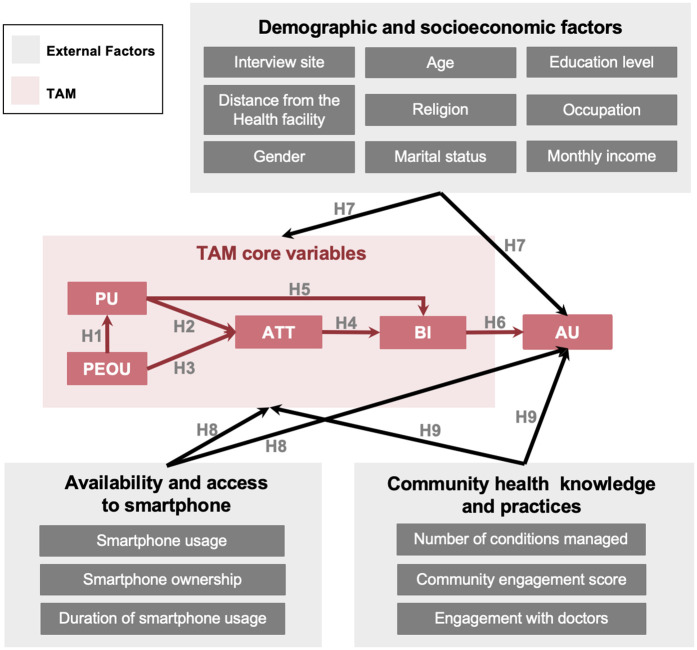
Graphical abstract of the conceptual framework of this study.

TAM consists of 4 major variables: perceived usefulness (PU), perceived ease of use (PEOU), attitude (ATT) and behavioral intention (BI), as shown in Fig 1. The definitions of these 4 factors are summarized in Definition of TAM variables.

### Description of TAM core variables and Actual Usage (AU) score

#### Perceived usefulness (PU).

PU was defined as the degree to which a person believes that a particular system would enhance his or her job performance [58]. According to TAM, if CHWs perceive a positive use-performance relationship with digital health tools, then they intend to use them for community health service provision. Perceived Usefulness directly predicts Attitude and Behavioral Intention to use the technology according to TAM.

#### Perceived ease of use (PEOU).

Davis defines PEOU as the degree to which a person believes that using a particular system would be free from effort [58]. Research has established PEOU as an important variable for the acceptance and usage of information technologies [59]. As a CHW’s’s perceived ease of using digital community health tools increases, the attitude towards them increase. Therefore, PEOU is a moderator of the relationship between attitude and behavioral intention.

#### Attitude (ATT).

Davis defined Attitude as the degree of evaluative effect that an individual attaches to using a system in his or her job [58]. Attitude is a CHW’s positive or negative behavior towards adopting digital community health tools [60]. CHWs who perceive positive outcomes from digital community health tools should express good attitudes towards them. According to TAM, attitude is a direct predictor of behavioral intention.

#### Behavioral intention (BI).

In this study, BI is CHW’s subjective probability that he/she will use digital community health tools [49]. According to TAM, Behavioral Intention is predicted to translate into actual usage of the technology, notwithstanding the availability of counteracting evidence [61].

#### Actual Usage (AU).

The actual usage level of digital tools (smartphones/ digital health tools) in community health management measured through 7 indices explained below:

(1) Have used digital tools in the work as a CHW. (yes/ no)
(2) Have handled diseases with digital tools. (yes/ no)
(3) Have taken and shared pictures with digital tools. (yes/ no)
(4) Have called doctors or ambulances with digital tools. (yes/ no)
(5) Have chatted with doctors over digital tools. (yes/ no)
(6) Have added diagnosis records of patients into digital tools. (yes/ no)
(7) Have searched and retrieved past patients’ records digital tools. (yes/ no)

AU scores were defined as a sum of the scores of these 7 indices.

### Definition of other variables

#### External factors.

TAM is designed as a 3-stage hypothetical process where external factors trigger cognitive functions of PEOU and PU, which in turn lead to an effective response (ATT and BI), hence influencing actual usage behavior [59]. The TAM central variables mediate the relationship between external factors and actual usage of digital health technologies. In other words, external factors here refer to exogenous variables that lie outside the core TAM constructs but may influence them.

Among the external factors we assessed included demographic and socioeconomic factors, availability and access to smartphones and community health knowledge and practices (Table 1). Among the demographic factors, age, gender and race have been inconsistently found to influence TAM variables and actual usage of the technology [62,63]. On the other hand, the socioeconomic factors of education, income, urbanization, and occupation have been inconsistently found to have varying degrees of influence on TAM and actual usage of digital health tools [64–69]. Given that, we could not rule out the possibility that those factors represent antecedent conditions affecting technology perception and use, for example, rather than internal psychological constructs like attitude or intention, which motivated us to include these external factors.

**Table 1:** External factors used in this study.

Name of external factors		Definition
**Demographic and socioeconomic factors**		Basic background information of each CHW such as *Interview site*, *Distance*, *Gender*, *Age*, *Religion*, *Marital status*, *Level of education, Occupation,* and *income*. Definition of each index is explained in Table 3 and Result section (“Background information of interviewees”> “Demographic and socioeconomic characteristics”).
**Availability and access to smartphones**	Smartphone usage	Ever used a smartphone or not. (yes/no)
	Smartphone ownership	The status of the ownership of one’s smartphone. (none/ personal/ shared)
	Duration of smartphone Usage	The duration of one’s smartphone usage. (months)
**Health care knowledge and practices**	Number of conditions Managed	The number of common diseases and other community healthcare related cases managed by each CHW. (number of cases)
	Community engagement Score (Derived from ICCM manual [Integrated Community Case Management manual developed by the World Health Organization and UNICEF to guide community-level diagnosis, treatment, and referral practices.], refined by capabilities of digital tools, and CHW responses)	Ever engaged in the major community health services. (1) Participating in Malaria prevention campaigns. (yes/ no) (2) Participating in HIV prevention campaigns. (yes/ no) (3) Encouraging hospital-based delivery. (yes/ no) * 1 for “yes”/0 for “no”. Min score = 0, max score = 3.
	Engagement with doctors (Derived from ICCM manual, refined by capabilities of digital tools, and CHW responses)	Variety of information one usually shares with doctors and other advanced medical support systems. (number of categories of information) (1) Sharing patient demographic information. (yes/no) (2) Sharing patient medical information. (yes/no) (3) Sharing patient management details. (yes/no) * 1 for “yes”,/0 for “no”. Min score = 0, max score = 3

### Conceptual framework

#### TAM testing hypotheses.

We set 6 hypotheses based on TAM, as shown in Fig 1.

**Hypothesis 1 (H1)** Perceived ease of use of digital health tools has a direct significant positive influence on Perceived usefulness by CHWs.

**Hypothesis 2 (H2)** Perceived usefulness of digital health tools has a direct significant positive influence on CHWs’ Attitudes towards them.

**Hypothesis 3 (H3)** Perceived ease of use of digital health tools has a direct significant positive influence on CHWs’ Attitudes towards them.

**Hypothesis 4 (H4)** Attitude towards digital health tools has a direct significant positive influence on CHWs’ Behavioral intention to use them.

**Hypotheses 5 (H5)** Perceived usefulness of digital tools has a direct significant positive influence on CHWs’ Behavioral intention to use them.

**Hypothesis 6 (H6)** CHWs’ Behavioral intention to use digital health tools positively and significantly influences the Actual usage of the tools.

#### TAM-external factors hypotheses.

We also developed the following hypothesis between the interaction of the external factors and TAM variables and the actual usage of digital health tools among CHWs as shown in Fig 1. Definitions of the external factors used in this study is summarized in Table 1.

**Hypothesis 7 (H7)** Demographic and socioeconomic factors such as interview site, distance from the nearest health facility, age, gender, religion, marital status, level of education, occupation and income level could significantly influence the TAM core variables and Actual usage of technology among CHWs.

**Hypothesis 8 (H8)** Availability and access to smartphones significantly influence the TAM core variables and Actual usage of technology among CHWs.

**Hypothesis 9 (H9)** Healthcare knowledge and practices could significantly influence the TAM core variables and Actual usage of technology among CHWs.

## Methods

### Research design

This was a cross-sectional, field-based mixed methods study in which access, knowledge, experience, and TAM constructs for digital health integration into community health systems were explored by interviewing CHWs. The study focused on digital health aspects relevant to community health services in most low-income settings, which are mobile health apps embedded with electronic community health records, telemedicine, digital dashboards and community surveillance capabilities.

### Research site and settings

Buikwe district is 37 km east of the capital Kampala. It is located on the northern shore of Lake Victoria, with a population of 422,771 people. It is the model district for the development, piloting and implementation of digital community health systems in Uganda. Over 5 digital health tools have had a footprint in the district, with varying success rates [30,70–73]. It is fundamentally a rural district, with urban and semi-urban clusters. Kiyindi and Bufumbe are semi-urban settlements in this district while Buwoola and Kinoni are rural communities.

Namuwongo is an urban slum settlement of 20,000 people served by a health Center III supported by a network of community health workers. It has also had a good footprint of digital community health tools [74]. Fig 6 shows the location of each research site.

**Fig 2:**
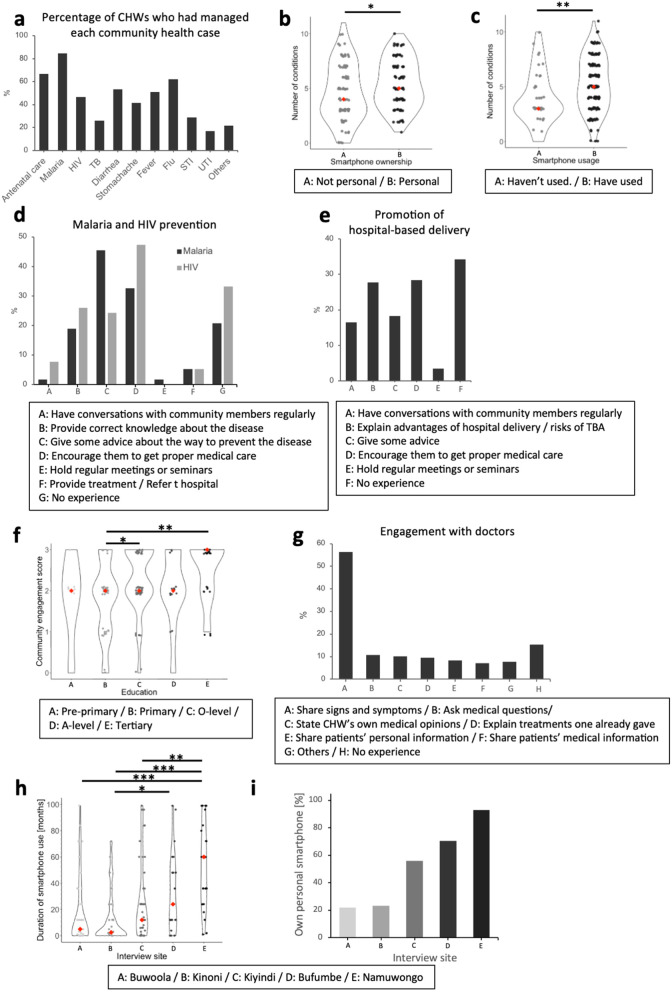
External factors associated with CHWs’ technology acceptance and usage. Panels (a–c) show how CHWs’ clinical engagement varies by smartphone access and usage. a: The graph illustrates the percentage of CHWs who had experienced the management of each community health case. b: CHWs who have their own smartphones had managed significantly higher number of conditions. (Mann-Whitney U test, p = 0.030*). c: CHWs who have used smartphones had managed significantly higher number of conditions. (Mann-Whitney U test, p = 0.007**). Panels (d–f) illustrate CHWs’ community engagement activities and their association with education levels. d: The graph shows the percentage of CHWs who had engaged in Malaria and HIV prevention through the exemplified methods within the box **(A to F)**. e: The graph shows the percentage of CHWs who had engaged in the promotion of hospital-based delivery through the exemplified methods within the box **(A to E)**. f: The level of education significantly influenced the Community engagement score (Kruskal-Wallis test followed by Dunn’s test, B-C: p = 0.030*, B-E: p = 0.008**). Panels (g–i) depict CHWs’ interaction with advanced medical support and smartphone availability. g: The graph shows the percentage of CHWs who had interacted with doctors in the exemplified ways within the box **(A to G)**. h: The duration of smartphone possession significantly differed among interview sites. (Kruskal-Wallis test followed by Dunn’s test, A-E p < 0.001***/B-E p < 0.001***/C-E p = 0.006**/B-D p = 0.020*). i: The graph shows the percentage of CHWs owning their personal smartphones in each interview site.

**Fig 3:**
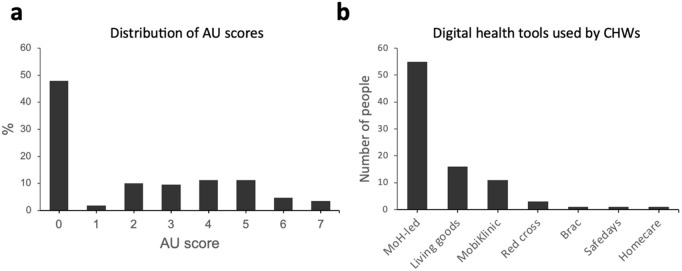
Actual usage of digital health tools among CHWs. a: The distribution of Actual usage (AU) scores (definition explained in Theoretical framework and hypothesis development section). b: The distribution of the number of CHWs using each existing digital health app. MoH-led digital health tools were the most major tools used among the interviewed CHWs.

**Fig 4:**
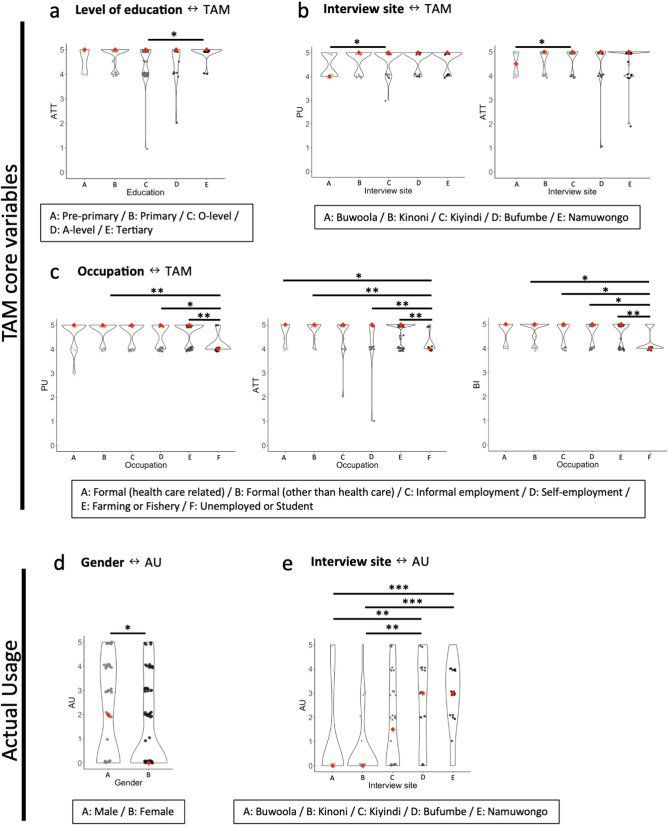
Demographic and socioeconomic factors and TAM, AU scores. The graphs show the results of analyses between Demographic and socioeconomic factors and TAM core variables. The black outlines in the background indicate the distribution of data points within each group. Only the results which included statistically significant pairs are shown. a: Level of education significantly influenced ATT scores. (Kruskal-Wallis test followed by Dunn’s test, p = 0.036*); b: Interview sites significantly affected PU and ATT scores. (Kruskal-Wallis test followed by Dunn’s test, left: p = 0.030*, right: p = 0.042*); c: Occupation had significant effects on PU, ATT and BI scores. (Kruskal-Wallis test followed by Dunn’s test, left: B-F p = 0.003**/D-F p = 0.010*/E-F p = 0.001**, middle: A-F p = 0.032*/B-F p = 0.001**/D-F p = 0.009**/E-F p = 0.001**, right: B-F p = 0.028*/C-F p = 0.046*/D-F p = 0.049*/E-F p = 0.005**); de: The graphs shows the results of analyses between Demographic and socioeconomic factors and AU scores. Only the results which included statistically significant pares are shown. d: AU scores showed significant difference between male and female. (Mann-Whitney U test, p = 0.038*); e: Interview sites significantly affected AU scores. (Kruskal-Wallis test followed by Dunn’s test, A-E p < 0.001***/B-E p < 0.001***/A-D p = 0.001**/B-D p = 0.003**).

**Fig 5:**
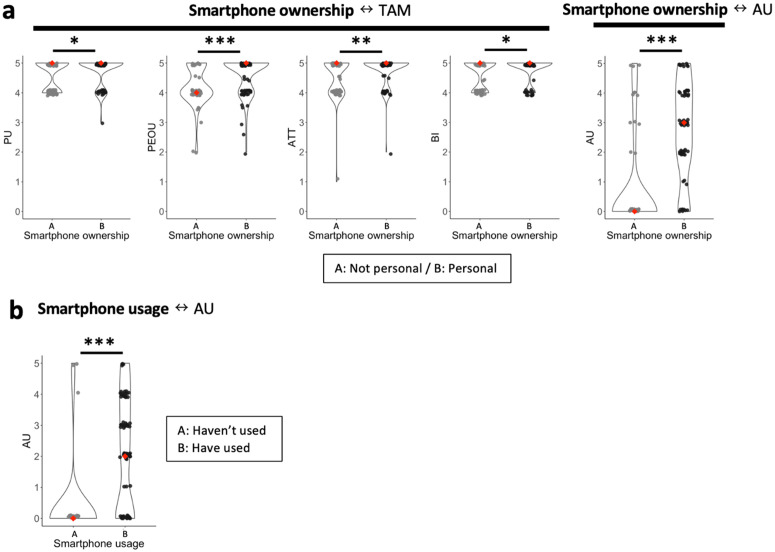
Availability and access to smartphones and TAM, AU scores. The graphs show the results of analyses between demographic and socioeconomic factors and the TAM core variables. The black outlines in the background indicate the distribution of data points within each group. a: CHWs who owned their personal smartphones showed significantly high scores in all TAM core variables (PU, PEOU, ATT and BI) and AU. (Mann-Whitney U test, PU: 0.043*/PEOU: p < 0.001***/ATT: p = 0.002**/BI: p = 0.038*/AU p < 0.001***). b: CHWs who had used smartphones showed significantly high AU score. (Mann-Whitney U test, p < 0.001***).

**Fig 6:**
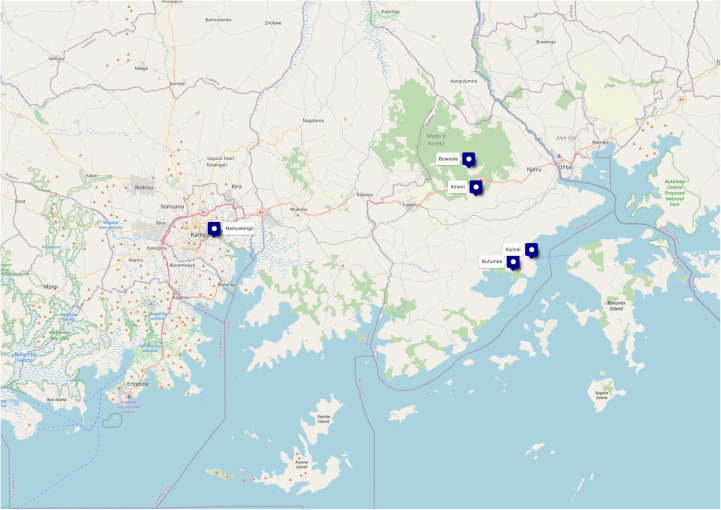
Location of the research sites (Contains information from OpenStreetMap (Source: https://umap.openstreetmap.fr/en/map/interview-sites_1292314, which is made available under Open Data Commons Open Data License https://opendatacommons.org/licenses/odbl/ that allows the distribution of produced work under the CC BY license.)).

### Study population

The study population was community health workers who had had some degree of exposure to digital health tools through training or outright usage in day-to-day community health service provision. This is because the selected sites, Buikwe District and Namuwongo in Kampala, had either previously implemented digital community health programs, as mentioned above, or provided to such technologies through urban community settings.

### Sample size, sampling procedure and recruitment

We used snowball and convenience sampling to recruit CHWs who were undergoing or had undergone the digitization process in Buikwe district and Namuwongo slum in Kampala. We invited the CHWs through their parish coordinators. CHWs from Buikwe were invited through the parish coordinators of Kiyindi and Bufumbe in Najja Subcounty and Kinoni and Buwoola in Najjembe Division. Interviews were conducted at mother health facilities such as Makonge Health Centre III, Bufumbe Muslim Health Centre II or nearby community spaces such as Buwoola C/U Primary School, Kinoni RC primary school in Najjembe and Muyenga Community Hall in Kampala, where they were interviewed after obtaining a written informed consent. We interviewed all available CHWs in each study site and nearby communities, bringing the total to 170. Out of the 903 CHWs in Buikwe District, 142 (15.7%) were interviewed. We interviewed 28 CHWs from Namuwongo and surrounding areas that report to Kisugu HCIII. This participation rate was primarily due to the fact that most CHWs are not full-time employees but serve voluntarily or on a part-time basis, making scheduling interviews challenging within a limited time frame. Nevertheless, participants were drawn from all major subcounties and community clusters, ensuring that the sample reflected the demographic and socioeconomic diversity of CHWs within the study areas.

### Ethics and consent

Ethical approval was sought and obtained from Makerere University, School of Health Sciences Research and Ethics Committee (Ref: MAKSHSREC-2022–424), where the first author (M.C.) was affiliated at the time of study design and data. Written informed consent was obtained from all participants prior to their involvement in the study. All methods were carried out in accordance with relevant guidelines and regulations such as the Declaration of Helsinki, as guided by the Makerere University School of Health Sciences’ Research and Ethics Committee.

### Instrument development and data collection tool

A semi-structured questionnaire was administered to CHWs. The questionnaire was composed of the following 5 sections (Section A – Section E).

#### Section A.

The first section collected background demographic information such as age, marital status, employment and residency.

#### Section B.

The second section explored the knowledge and practices of CHWs in community health service provision. This collected information on the number and nature of diseases managed in the community per month, the level of community engagement, and knowledge and interaction with advanced support systems. The subsection of community engagement inquired about CHW strategies with community members in regard to the prevention of common diseases (malaria and HIV) and the transition from traditional birth attendants to facility-based delivery. These were open-ended questions. To understand CHWs’ level of interaction with advanced support systems, we asked them if they had ever consulted or referred patient cases. We then probed them for the information they routinely share with doctors during referral or routine consultation to understand the quality of these interactions.

#### Section C.

Section C explored knowledge, access and usage of smartphones for community health services. This explored smartphone ownership, history of smartphone usage, duration of smartphone usage, usage of mobile health tools and challenges with digital health.

#### Section D.

Section D explored TAM variables among CHWs by collecting data about PU, PEOU, ATT, and BI.

### Measuring TAM variables

To measure the TAM variables, different scales were adopted from the existing TAM literature and modified to fit the study subject. To quantify the constructs, a 5-point Likert scale was created for each response. The answer options ranged from 1 (strongly disagree) to 5 (strongly agree). A total of 16 items were used to measure the 4 TAM core variables. 4 each for PU and PEOU, 2 for ATT and 6 for BI as indicated in (Table 2).

**Table 2:** Definition of subindices to measure TAM variables.

Subindex	Definition
**PU (Perceived Usefulness)**	
PU1	Digital tools create time convenience.
PU2	Digital tools bring about information efficiency.
PU3	Digital tools come with financial convenience.
PU4	Digital tools enhance connectivity in community health programs.
**PEOU (Perceived Ease Of Use)**	
PEOU1	Learning to operate Digital tools has been easy for me.
PEOU2	I find it easy to get Digital tools to do what I want it to do.
PEOU3	It is easy for me to become skillful at using Digital tools.
PEOU4	I found Digital tools easy to use.
**ATT (Attitude)**	
ATT1	I think using Digital tools is a good idea.
ATT2	I think using Digital tools is interesting.
**BI (Behavioral Intention)**	
BI1	I am very likely to use Digital tools if it is available.
BI2	I have the intention to use Digital tools routinely.
BI3	I will recommend other CHPs to use the MobiKlinic tool.
BI4	I am willing to learn the usage of an mHealth tool.
BI5	I am willing to incur financially to support usage of the digital health tool.
BI6	I am willing to apportion time to learn and use the digital health tool.

#### Section E.

Section E measured the actual usage of digital health tools among CHWs. To assess the Actual usage (AU) level of digital tools such as smartphones in their work as a CHW, we identified 7 mHealth features common in community health digital platforms. These features were systematically aggregated from five widely used digital health apps deployed by the Ministry of Health/Malaria Consortium, Living Goods, BRAC, MobiKlinic, and the Red Cross. The selection was based on functional overlap across these tools, representing the most essential and recurrent digital health activities among CHWs, such as recording patient data, communicating with healthcare professionals, and retrieving patient information. We asked CHWs if they had used each of these features, and we tallied up each individual’s responses (true for 1, false for 0) and obtained a summation.

### Data collection

Each CHW was interviewed using the tool described in. CHWs were invited to their respective mother health centers and other community spaces where they were interviewed by the research team with the help of well-trained research assistants (RAs). Responses were recorded on paper forms that were checked for completeness. Additional phone calls were made by RAs to fill out critical missing information. A total of 170 interviews were considered for analysis.

### Data management

Data were entered into a designed Open Data Kit (ODK) collection form linked to an encrypted Google spreadsheet. The complete dataset was downloaded as a Microsoft Excel spreadsheet to perform cleaning, categorization of open-ended responses and any other data reduction. Open-ended responses such as occupation, community engagement and engagement with doctors were deductively fit into preset categories that were matched with numerical representations. Likert scale data were also assigned representative numerical values. The data were then transferred into SPSS version 28 for correlation and regression analysis.

### Data analysis

Cross-tabulation was performed to obtain frequencies, means, and standard deviations among other basic statistics using SPSS version 28. SPSS 28 was also used to conduct reliability tests on TAM construct data in addition to correlation and regression analysis. Cronbach’s alpha values were benchmarked for reliability testing of the data in the TAM core variables of PU, PEOU, ATT and BI.

TAM variables were transformed into representative median Likert scale values for correlation analysis between themselves and with external factors, AU and other outcomes. Correlation analysis was performed between the core variables of the TAM model, i.e., PU, PEOU, ATT and BI. Kendall’s Tau b correlation coefficient was obtained for TAM-TAM and TAM-Continuous external factor relationships. A Pearson correlation coefficient was obtained between continuous external factors like age, and AU. Kruskal Wallis or Mann-Whitney U tests were also performed between TAM variables and AU, and external factors where applicable. Each model included one external factor at a time to prevent multicollinearity and overfitting. Where significant results were observed, we applied a post hoc test with multiple comparison adjustments (i.e., Bonferroni correction) to control for Type I error.

Open-ended responses about referral medical system engagement were inductively analyzed to exhaust the representative codes as shown in Fig 2g. Three themes were deduced from these codes to resonate with the CHWs’ scope of work as shown in Table 1. Each response was checked for the presence of these 3 themes. Participation in malaria and HIV prevention and hospital delivery campaigns were used to measure community engagement. A score of 1 was given for each item code present in the response, and a summation was obtained for each interview.

## Results

### Background information of interviewees

#### Demographic and socioeconomic characteristics.

We obtained 170 responses that were fit for analysis. Of these, 115 (67.6%) were women. The average age was 37.9 years, 42.9% had obtained at most an ordinary level education certificate (i.e., O-level, equivalent to the 10th Grade), and most of them were married (68.2%). The majority of CHWs were involved in primary production (e.g., farming, fishing) (38.5%), while their monthly incomes varied from a minimum of 20,000 UGX to a maximum of 1,500,000 UGX, with an average of 209,006.5 UGX and median of 200,000 UGX (approximately 1 USD = 3,700 UGX at the time of the study). Distances from the nearest health facility also varied from minimum of 0.5 km to maximum of 20 km (average ≒ 3.3 km). The overall characteristics are summarized in Table 3.

**Table 3:** Socio-economic characteristics and smartphone access of the interviewees.

Variable	N (%)
**Interview site**	
Buwoola	41 (24.1)
Kinoni	30 (17.6)
Kiyindi	44 (25.9)
Bufumbe	27 15.9)
Namuwongo	28 (16.5)
**Gender**	
Male	55 (32.4)
Female	115 (67.6)
**Age**	
Under 19	8 (4.7)
20–29	39 (22.9)
30–39	53 (31.2)
40–49	41 (24.1)
50–59	18 (10.6)
Over 60	11 (6.5)
**Religion**	
Catholic	58 (34.1)
Protestant	27 (15.9)
Pentecost	52 (30.6)
Muslim	28 (16.5)
Others	5 (2.9)
**Marital status**	
Married	116 (68.2)
Never married	34 (20.0)
Divorced	10 (5.9)
Widowed	10 (5.9)
**Level of education**	
Preprimary	9 (5.3)
Primary	44 (25.9)
O-level	73 (42.9)
A-level	20 (11.8)
Tertiary	24 (14.1)
**Occupation**	
Formal employment (Health care related)	16 (9.5)
Formal employment (Other than Health care)	22 (13.0)
Informal employment	27 (16.0)
Self-employment	27 (16.0)
Farming/Fishery	65 (38.5)
Unemployed/Student	12 (7.1)
**Past experience of smartphone use**	
Yes	134 (78.8)
No	36 (21.2)
**Current ownership of smartphones**	
Personal	85 (50.0)
Sharing	13 (7.6)
Other (Stolen, Technical issue, etc.)	33 (19.4)
Not having	39 (22.9)

### Community health care knowledge and practices

#### Management of common diseases.

A significant majority (n = 165, 97.1%) had at least had a patient interaction in their communities in the past month. On average, each CHW had handled 5 community cases the previous month. There were variations in the types of cases handled by CHWs, as shown in Fig 2a. More than half of the participants mentioned having interfaced with a case of malaria (84.6%), flu (62.1%), diarrhea (53.3%) and fever (50.9%), while only 29 (17.6%) had interfaced with UTIs. There were significant positive correlations between duration of smartphone usage (B = 0.282, P < 0.001) with the number of diseases managed by CHWs per month. Additionally, those who had ever used smartphones and those who owned their personal smartphones showed significantly higher numbers compared to others (Figs 2b and 2c).

### Community engagement for better health

#### Prevention of malaria and HIV.

The majority (79.3% and 66.9%) of CHWs indicated participating in malaria and HIV prevention community engagement activities, respectively (Fig 2d). Almost half of CHWs (46.5%) shared knowledge on the prevention of malaria as shown in (Fig 2d). For HIV prevention, 47.3% of them encouraged HIV-positive patients to seek comprehensive and quality management from qualified health facilities (Fig 2d). A small proportion (5.3%) of CHWs had availed some sort of treatment or referral for HIV and malaria patients in the community during community engagement (Fig 2d).

#### Promotion of hospital-based delivery.

As indicated in, the majority of CHWs (65.4%) had participated in community engagement to encourage hospital-based delivery the previous month (Fig 2e). A total of 27.8% explained the advantages of hospital delivery or the risks of Traditional Birth Attendant (TBA), and 28.4% encouraged pregnant women to seek proper medical care from health facilities. A total of 17.2% of CHWs maintained contact with pregnant women by visiting their homes regularly (Fig 2e). A total of 3.5% of them held regular meetings or seminars to provide them with correct information (Fig 2e). There was a significant positive correlation between experience of smartphone usage (B = 0.219, P < 0.005) and attainment of an ordinary level education certificate (Fig 2f) with the community engagement score (definition: Table 1).

#### Utilization of advanced medical support structures.

The Ministry of Health and community health implementing partners, such as non-governmental organizations and international agencies that support community health service delivery, had devised means for CHWs to consult or refer patients with ailments beyond their scope. A total of 26 (15.3%) of the CHWs had not sought or received advanced medical support for their patients in the previous month (Fig 2g). Most CHWs (56.5%) preferred to share the signs and symptoms of their patients, while 10.5% asked medical questions from supportive healthcare practitioners as indicated in Fig 2g. These variations partly reflect differences in the functionalities of mHealth tools available across implementing partners. In many cases, CHWs use digital tools primarily during referrals, where communication with healthcare professionals tends to be unidirectional, focusing on transmitting patient information. Besides that, there was a significant positive correlation between the AU scores and the extent of the engagement with doctors (definition: Table 1) (B = 0.185, P < 0.05).

### Access to and usage of smartphones

#### Access to smartphones.

A significant majority (78.8%) of CHWs had ever used a smartphone. At the time of the interview, 98 (57.6%) CHWs had access to a smartphone, which is almost thrice the national average smartphone penetration rate of 21%. A total of 67.3% of these smartphones were accessed through the Ministry of Health or implementing partners like Malaria Consortium. Among those who had access to smartphones, 86.7% had personal smartphones, and the rest were sharing with acquaintances (Table 3), and the average duration of smartphone usage was 35.7 months (median = 24 months; SD = 29.9 months). Access to smartphones varied among interview sites. CHWs who were interviewed from Bufumbe in Najja Subcounty and Namuwongo in Kampala were more likely to possess personal smartphones and tended to show longer duration of smartphones usage compared to their counterparts (Figs 2h and 2i).

### Technology acceptance and actual usage among CHWs

#### Reliability test results.

We calculated Cronbach’s alpha to check the internal consistency of each core TAM variable and results are shown in Table 4. The Cronbach’s alpha was greater than 0.7 for PEOU, ATT and BI (Table 4; PEOU: 0.831, ATT: 0.972, BI: 0.846). For PU, Cronbach’s alpha was slightly below 0.7 (PU: 0.695), which is acceptable for the level of research [75]. Based on these results, all the TAM factors are considered to have good internal consistency.

**Table 4:** The distribution of each TAM sub-index and the results of Cronbach α calculation.

TAM core variables		Mean	S.D.	Cronbach α
PU	PU1	4.681	0.506	0.695
	PU2	4.681	0.574	
	PU3	4.368	0.875	
	PU4	4.607	0.652	
PEOU	PEOU1	4.259	0.966	0.831
	PEOU2	4.425	0.732	
	PEOU3	4.360	0.843	
	PEOU4	4.453	0.773	
ATT	ATT1	4.671	0.577	0.972
	ATT2	4.671	0.577	
BI	BI1	4.706	0.457	0.846
	BI2	4.643	0.549	
	BI3	4.678	0.469	
	BI4	4.720	0.450	
	BI5	4.343	0.889	
	BI6	4.650	0.521	

#### Actual usage of digital tools in community health.

Table 5 shows the proportion of CHWs that used each of the mobile health features. 87 participants (51.5%) had used smartphones in community health service provision, while 66 (39.1%) had managed a disease condition using custom mobile health tools. AU scores were defined as a sum of AU sub-indices scores. Fig 3a shows the distribution of these scores. The mode scores were 4 and 5 features, which were used by 11.2% of CHWs each. Calling was the most common means of reaching Advanced Medical Support (Mean=0.314, S. D =0.465). Of the 66 that had used custom mobile health tools, 55 (83.3%) had used MoH-led platforms, 16 Living Goods platforms, 11 MobiKlinic Platforms, 3 Red Cross platforms, and 1 each for Brac, SafeDays^R^, and Homecare (Fig 3b).

**Table 5:** Distributions of actual usage sub-indices.

Sub-indices of Actual usage score	Yes (n/ %)	No (n/ %)
AU1: Have used digital tools in the work as a CHW.	87 (51.5)	82 (48.5)
AU2: Have handled diseases with digital tools.	39 (23.1)	130 (76.9)
AU3: Have taken and shared pictures with digital tools.	53 (31.4)	116 (68.6)
AU4: Have called doctors or ambulances with digital tools.	39 (23.1)	130 (76.9)
AU5: Have chatted with doctors over digital tools.	33 (19.5)	136 (80.5)
AU6: Have added diagnosis records of patients into digital tools.	29 (17.2)	140 (82.8)
AU7: Have searched and retrieved past patients’ records with digital tools.	66 (39.1)	103 (60.9)

### TAM hypotheses test results

According to the correlation analysis (Table 6), H1 through H5 supported TAM predictions of technology acceptance. Perceived usefulness had the greatest individual influence on CHWs’ behavioral intention to use digital health tools, as indicated in Table 6.

**Table 6:** Results of correlation analyses among TAM factors.

Hypothesis (n)	Construct	Kendall Tau b (p)
H1 (140)	PEOU ⇔ PU	0.493 (< 0.001)
H2 (161)	PU ⇔ ATT	0.713 (<0.001)
H3 (139)	PEOU ⇔ ATT	0.588 (< 0.001)
H4 (149)	ATT ⇔ BI	0.848 (< 0.001)
H5 (148)	PU ⇔ BI	0.737 (< 0.001)
H6 (151)	BI ⇔ AU	0.038 (0.447)

### TAM–external factors hypotheses test results

Table 7 and Figs 4 and 5 summarize the results of analyses between external factors and TAM/AU scores. Specifically, Table 7 shows correlation analyses performed for continuous or ordinal variables. For categorical demographic variables such as gender and education, Figs 4 and 5 show their relation to TAM/AU scores.

**Table 7:** Summary of the results of the correlation analyses between TAM/ AU and External factors.

External factors (test name)		TAM core variables and Actual usage (Coefficient, p-value, n)				
		PU (*K*)	PEOU (*K*)	ATT (*K*)	BI (*K*)	AU (*P*)
**Demographic and socioeconomic factors** (Hypothesis 7)	**Age (K)**	0.141, 0.027^*^, 164	-0.097, 0.142, 141	0.099, 0.120 164	0.085, 0.202, 151-	0.035, 0.545, 169-
	**Distance (K)**	-0.193, 0.006^**^, 150	-0.279, 0.000^**^, 131	-0.160, 0.022^*^, 150	-0.066, 0.373, 138-	-0.158, 0.013^*^, 155
	**Income (K)**	0.061, 0.384, 149	0.048, 0.511, 126-	0.008, 0.905, 150	-0.007, 0.920, 138	0.119, 0.059, 154
**Availability and access to smartphones** (Hypothesis 8)	**Duration (months)**	0.060, 0.359, 164	0.067, 0.330, 141-	0.138 0.038^*^ 164	0.091, 0.190, 151	0.527, 0.000^**^, 169
**Health care knowledge and practices** (Hypothesis 9)	**Number of conditions managed**	0.225, 0.001^**^, 164	0.097, 0.161, 141	0.263, 0.000^**^, 164	0.210, 0.000^**^, 151	0.261, 0.001^**^, 169
	**Community engagement score**	0.097, 0.179, 164	-0.069, 0.367, 141	0.124, 0.086, 164	0.108, 0.153, 151	0.219, 0.004^**^, 169
	**Engagement with doctors**	0.202, 0.006^**^, 164	0.005, 0.946, 141	0.259, 0.000^**^, 164	0.235, 0.002^**^, 151	0.247, 0.001^**^, 169

***K*** Kendall’s Tau B between TAM variables and external factors, ***P*** Pearson Correlation between actual usage and external factors, **n**-number of valid entries, * correlation is significant at 0.05, ** correlation is significant at 0.01.

#### Hypothesis 7.

Age, residency (Distance, Interview site), Level of education and Occupation influenced TAM core variables (Table 7, Fig 4a-4c). AU was also influenced by several factors such as residency (Distance, Interview site), Income and Gender (Table 7, Fig 4d and 4e). For residency, those who lived near the health facility, and who lived in Bufumbe and Namuwongo tended to show higher TAM and AU scores (Table 7, Fig 2b and 2e).

#### Hypothesis 8.

Those who own personal smartphones (Smartphone ownership) showed significantly higher scores in all TAM variables and AU scores, while Smartphone usage only influenced AU scores (Fig 5a and 5b). The longer CHWs used their smartphones (Duration), the higher their ATT and AU scores tended to be (Table 7).

#### Hypothesis 9.

Health care knowledge and practices were associated with better TAM rankings and AU scores (Table 7). An increase in the number of conditions managed by CHWs positively influenced PU (B=0.225, P=0.001), ATT (B=0.263, P<0.001), BI (B=0.210, P<0.001) and AU (B=0.261, p=0.001). CHWs who had higher community engagement score tended to show higher AU scores (B=0.219, p=0.004). Improved interaction with advanced medical support positively and significantly influenced PU (B=0.202, P=0.006), ATT (B=0.259, P<0.001), BI (B=0.235, P=0.002) and AU (B=0.247, p=0.001).

## Discussion

Our study obtained very high average scores for PU, PEOU, ATT and BI, revealing favorable views and intentions of CHWs towards using digital health technologies (cross ref). Most importantly, correlation analysis reveals that the digitization of the community health space follows the technology acceptance model, and that TAM is a good predictor of CHWs’ behavioral intention to use digital health tools. This is consistent with prior studies in both health information technology [54,55,76] and other sectors [49,50,53].

However, behavioral intention didn’t show significant influence on actual usage of digital health tools among CHWs, opening up possibilities of influence from other external factors. Among the external factors tested, smartphone ownership showed significant influence on each of the TAM variables and AU with history, and duration of smartphone usage also showing some influence on one or 2 of TAM/AU variables. This may indicate that smartphone possession is the missing link in translating the high BI into AU, considering that having personal smartphones rather than sharing with others can increase the accessibility to the device. Having easy-to-access personally owned smartphones would translate into familiarity to the device and smooth navigation of apps, including mobile health apps, hence accelerating acceptance and usage of digital health tools [77].

Among other external factors, residency (Interview site, distance from the nearest health facility) had significant effects on several TAM core variables and AU scores. Residing in urban and suburban settlements of Namuwongo and Bufumbe was a significant positive influencer of actual usage. This could be explained by the exposure to smartphones and technologies in general and better incomes in urban settings. Our results partly align with results from other studies and further highlight the role socioeconomic and demographic factors play in the adoption and actual usage of technology [65–69]. A negative, significant correlation between smartphone ownership and distance from health facilities indicates that either CHWs in close proximity to health facilities could be better digitized than their distant counterparts or health facilities are only in peri-urban settings where smartphone ownership is higher. With this, digitization seems to be further entrenching the rural-urban divide in health service delivery. On a positive note, the government and partners have been pro-active in the digitization journey as 67% of smartphones possessed by CHWs were procured by government and partners. Additionally, smartphone access among CHWs stood at 58% which is almost triple the national average of 21%. Digital readiness and affluence could have favored digitization among urban and peri-urban CHWs [77,78], but deliberate, affirmative government action for rural CHWs will be a prerequisite in optimal leverage of digital tools to achieve universal health coverage in 2030 [64,65].

Our results show that technology acceptance among CHWs doesn’t seem to be gender or age sensitive except that older CHWs find technology more useful than their younger counterparts which could be a testimony to their long experience of using the cumbersome paper-based records management systems, an experience they hoped would be improved by the technology [35,79].

There was a significant positive correlation between actual usage of digital tools and community health knowledge and practices (Number of conditions managed, Community engagement scores, and Engagement with doctors). Additionally, an increase in the number of conditions managed and improved level of engagement with doctors were associated with positive influences on the TAM variables PU, ATT, BI and AU and vice versa. Smartphone usage improved community engagement because smartphones improve information and communication efficiency and smooth interactions with the community and advanced medical support systems [22,24,35]. On the other hand, CHWs that managed more conditions and consulted more often found the technology useful because it reduced their workload by eliminating paper records taking and enhancing communication and general data management [35,54]. This would consequently influence their attitudes and behavioral intentions positively. Additionally, smartphones improve CHWs’ community standing and stimulate community interest in CHWs’ work [22,80]. This results in increased interaction with community members.

At the same time, we have to note that potential confounding by demographic and socioeconomic factors on TAM variables cannot be ruled out. Future studies should incorporate multivariate models to adjust for these factors and further validate the independent effects of smartphone ownership on technology acceptance and usage. Additionally, conducting a subgroup analysis limited to CHWs with personal smartphones may provide more in-depth insights, as our findings suggest that smartphone access has a strong influence on the relationship between behavioral intention and actual usage. Future studies can examine this association among CHWs with guaranteed access to digital tools to better understand how behavioral intention translates into real-world digital health use.

Lastly, a higher proportion of CHWs engaging in malaria campaigns compared to HIV prevention and safe delivery campaigns may indicate a high prevalence of malaria, or technology skepticism by community members regarding sensitive information shared in prevention and management of HIV and pregnancy. If the latter is true, then it highlights a need for further community engagement regarding technology usage in healthcare, and strengthening of data regulatory frameworks to reassure individuals of personal data safety. Our findings come at a critical time when many governments in low-income countries are rolling out National Community Health Digitization Strategies [1,8,13]. While training and motivation have previously been identified, and remain vital prerequisites for digitization, our results highlight that adoption and sustained use also hinge on broader structural factors—particularly sustainable smartphone procurement models, equitable digital infrastructure, and deliberate support for rural and lower-resourced CHWs. The subgroup differences observed in urban versus rural settings, and across age groups, underscore that adoption strategies cannot be “one size fits all,” but must be tailored to socioeconomic and contextual realities. The insights from this study are relevant to other low- and middle-income countries where similar disparities in digital readiness exist. By empirically demonstrating the role of external factors such as smartphone ownership and urbanization in shaping technology acceptance, our work reinforces the applicability of the TAM framework in community health contexts and contributes original evidence to inform equitable digitization strategies across LMICs.

## Conclusions

According to our study, CHWs’ behavior intention to use digital health technology conforms to the TAM hypothesis but smartphone availability is essential to translate the good behavioral intention into actual routine use of digital health tools in community health service provision. Therefore, effort should be undertaken to ensure universal access to smartphones by CHWs especially in more remote areas that would derive where community members would derive maximum benefit from digitization of their CHWs.

Any digitization drive should be sensitive to prevailing demographic and socioeconomic fissures by conducting thorough needs assessment of CHWs and their communities, putting into consideration age, income, gender and urbanization disparities, to a ensure smooth, but equitable digitization of the community health space.

Continuous active community engagement coupled with strengthened, and practical personal data regulatory frameworks will allay digitization fears among community members, improve their engagement with CHWs and smoothen the digital health transition.

In this study, we discovered a positive feedback loop between access to smartphones, community health skills and TAM variables among CHWs. Access to a smartphone improves community health skills and efficiency, which in turn reinforces the TAM constructs, resulting in improved behavioral intention to use digital health tools. With the availability of a smartphone and associated support, adoption and routine use of digital health technology becomes self-limiting. Digital health tools that offer practical solutions to challenges faced by CHWs will enjoy accelerated adoption and use among CHWs and the reverse is true.

### Preprint

This study was previously posted as a preprint on Research Square [81].
